# A potential role for *Giardia* chaperone protein GdDnaJ in regulating *Giardia* proliferation and *Giardiavirus* replication

**DOI:** 10.1186/s13071-023-05787-0

**Published:** 2023-05-25

**Authors:** Hongbo Zhang, Chunyan Zhao, Xichen Zhang, Jianhua Li, Pengtao Gong, Xiaocen Wang, Xin Li, Xin Wang, Xu Zhang, Shuqin Cheng, Taotao Yue, Nan Zhang

**Affiliations:** Key Laboratory of Zoonosis Research, Ministry of Education, Institute of Zoonosis, College of Veterinary Medicine, Jilin University, Changchun, 130062 China

**Keywords:** *Giardia duodenalis*, *Giardiavirus*, RNA-dependent RNA polymerase, Chaperone protein

## Abstract

**Background:**

*Giardia duodenalis* (referred to as *Giardia*) is a flagellated binucleate protozoan parasite, which causes one of the most common diarrheal diseases, giardiasis, worldwide. *Giardia* can be infected by *Giardiavirus* (GLV), a small endosymbiotic dsRNA virus belongs to the Totiviridae family. However, the regulation of GLV and a positive correlation between GLV and *Giardia* virulence is yet to be elucidated.

**Methods:**

To identify potential regulators of GLV, we performed a yeast two-hybrid (Y2H) screen to search for interacting proteins of RdRp. GST pull-down, co-immunoprecipitation and bimolecular fluorescence complementation (BiFC) assay were used to verify the direct physical interaction between GLV RdRp and its new binding partner. In addition, their in vivo interaction and colocalization in *Giardia trophozoites* were examined by using Duolink proximal ligation assay (Duolink PLA).

**Results:**

From Y2H screen, the *Giardia* chaperone protein, *Giardia* DnaJ (GdDnaJ), was identified as a new binding partner for GLV RdRp. The direct interaction between GdDnaJ and GLV RdRp was verified via GST pull-down, co-immunoprecipitation and BiFC. In addition, colocalization and in vivo interaction between GdDnaJ and RdRp in *Giardia trophozoites* were confirmed by Duolink PLA. Further analysis revealed that KNK437, the inhibitor of GdDnaJ, can significantly reduce the replication of GLVs and the proliferation of *Giardia*.

**Conclusion:**

Taken together, our results suggested a potential role of GdDnaJ in regulating *Giardia* proliferation and GLV replication through interaction with GLV RdRp.

**Graphical Abstract:**

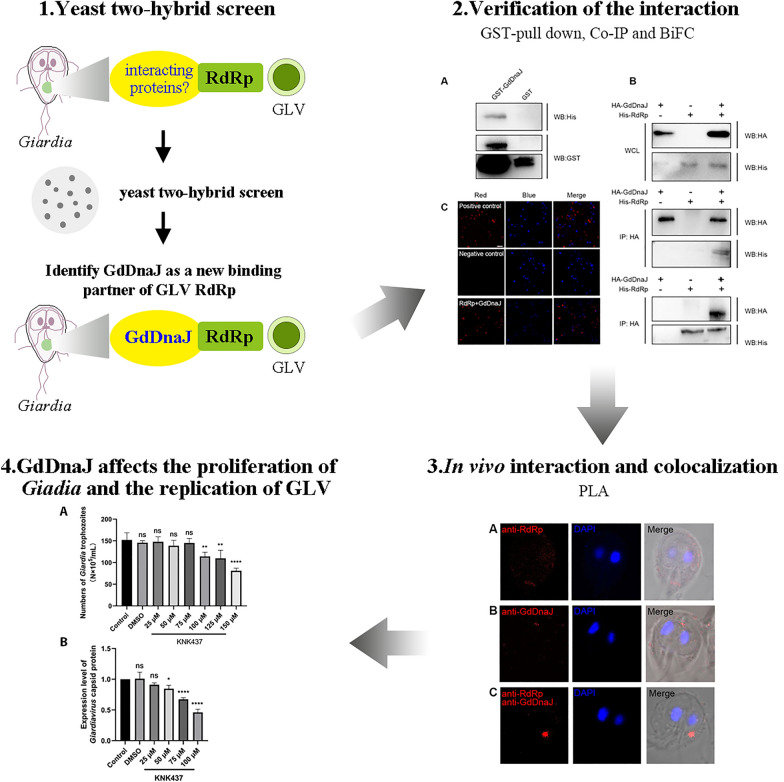

**Supplementary Information:**

The online version contains supplementary material available at 10.1186/s13071-023-05787-0.

## Background

*Giardia duodenalis* (formerly known as *Giardia lamblia*, referred to as *Giardia*) is one of the most prevalent protozoan parasites colonizing in the small intestine of humans and a wide range of animals [1–3]. It causes the most common enteric disease, called giardiasis, worldwide, with ~ 1 billion humans affected and ~ 183 million symptomatic cases reported each year, severely threatening human health [4–6]. *Giardia* has been included in the WHO’s Neglected Disease Initiative since 2004 [7, 8]. However, there is still a lack of desirable drugs and prevention methods regarding *Giardia* infections due to the unclear underlying pathogenesis mechanism.

*Giardia* has long been proposed as an intriguing and potentially powerful model organism owing to its evolutionary position and unique cellular structures [9]. It is regarded as a primitive eukaryote at the transition of prokaryotes and eukaryotes [10]. *Giardia* has two nuclei, which are both transcriptionally active, and has unusual ultrastructures that lack ‘typical’ eukaryotic organelles such as mitochondria, peroxisomes and a classic Golgi apparatus [6, 11]. In addition, the life cycle of *Giardia* is simple with only two stages, the proliferating trophozoite and the infectious cyst [3, 12]. Thereby, *Giardia* provides a good model system for studying many basic molecular and cellular evolutionary processes of eukaryotic cells.

Increasing evidence has shown that many parasites can be infected by viruses which can modulate the interaction of the parasites with their host and might have consequences on the pathogenicity and virulence of the parasites [13, 14]. For instance, *Leishmania* virus can activate the mouse TLR3 pathway through the virus dsRNA, which leads to the secretion of a large number of pro-inflammatory mediators that induce a persistent inflammatory reaction and promote the persistent infection of the parasite [15]. Additionally, *Trichomonas vaginalis* virus can stimulate the secretion of interferon regulatory factor 3 and type I interferon by activating TLR3 in human epithelial cells and promoting the inflammatory response [16]. *Giardia* can be infected by the *Giardia lamblia* virus (GLV), the only recognized species of genus GLV, and belongs to the *Totiviridae* family, which was originally identified in a *G. duodenalis* isolate from a human patient (HP-1, Human Portland-1) 35 years ago [14, 17, 18]. The growth of *Giardia* is stopped when GLV reaches a high titer (approximately 500,000 viruses per trophozoite) [19]. However, the effect of GLV on the virulence of *Giardia* is yet to be clarified.

GLV is a non-enveloped linear double-strand RNA (dsRNA) virus [19]. The genome of GLV is about 6.3 kb and contains two overlapping ORFs that encode two proteins, the viral capsid protein (100KDa) and RNA-dependent RNA polymerase (RdRp, 190KDa), respectively [18, 19]. It has been suggested that GLV enters trophozoites through receptor-mediated endocytosis and leaves the cell without lysis [20]. All these characteristics make it an intriguing model for studying the evolution of viruses and the relationship between viruses and protozoa. However, the regulation of GLV replication is largely unclear.

Given that little is known about the regulators of GLV replication, we decided to look for proteins that can interact with GLV RdRp, a key regulator of virus replication. Using a yeast two-hybrid (Y2H) screen, we identified a novel interaction between GLV RdRp and *Giardia* DnaJ (GdDnaJ), whose function is still unknown. Intriguingly, further analysis revealed that GdDnaJ affects the proliferation of *Giardia* and the replication of GLV potentially by interacting with GLV RdRp.

## Methods

### Parasites and cell culture

The *Giardia duodenalis* GdA1 strain that contains virus was cultured and maintained as previously described [21]. The HEK-293 T cells, obtained from the cell bank of the Chinese Academy of Sciences (China), were cultured and maintained in DMEM supplemented with 10% FBS.

### *Giardia* cDNA library construction

To generate the *Giardia* cDNA library, the *Giardia* trophozoite was prepared for total RNA extraction. The total RNA of *Giardia* trophozoite was extracted by Trizol (Invitrogen) and then reverse-transcribed into cDNA. The *Giardia* cDNA library was constructed using SMART cDNA Library Construction kit. The library contained about 3.7 × 10^6^ cfu independent recombinants, and the recombination rate of the library was 100%. The size of insertion fragments ranged from 0.4 kb to 2 kb.

### Y2H screen

For Y2H screen, truncation fragment (452–690 aa) of RdRp was amplified from *Giardia* cDNA and cloned into the bait vector pGBKT7 (Clontech Laboratories) to construct the pGBKT7-RdRp plasmid. The bait protein was successfully expressed in yeast cells without obvious toxicity and self-activation. Y2H screening against the *Giardia* cDNA library was performed by using the Gal-4-based Gold Yeast Two-Hybrid System (Clontech Laboratories) according to the user manual instructions. The identity of positive clones was determined by sequencing. The interaction between RdRp and the chaperone protein GdDnaJ was preliminarily verified by alpha-galactosidase assay.

### Plasmid construction

To generate the pET-32a-RdRp and pGEX-4 T-1-GdDnaJ fusion constructs used for GST pull-down experiments, RdRp and GdDnaJ were amplified and cloned into the pGEX-4 T-1 (GE Healthcare Life Sciences) and pET-32a (Novagen) vectors, respectively. For co-immunoprecipitation experiments, PCR amplifications of RdRp and GdDnaJ were introduced in frame into the respective pcDNA3.1-Myc-His (Invitrogen) and pcDNA3.1-HA vectors (Invitrogen) to generate the pcDNA3.1-Myc-His-RdRp and pcDNA3.1-HA-GdDnaJ plasmids. For bimolecular fluorescence complementation (BiFC) assay, RdRp and GdDnaJ were inserted into pbJUN-HA-KN151 vector between NheI and PvuI sites and pbFOS-Myc-LC151 vector between NheI and KpnI sites to construct pbFos-RdRp and pbJun-GdDnaJ plasmids, respectively. All the primers used in this study were designed by Oligo 7 software and are listed in Additional file 1: Table S1.

### GST pull-down

*Escherichia coli* Transetta competent cells (DE3) were transformed with either pET-32a-RdRp or pGEX-4 T-1-GdDnaJ plasmid (see above, plasmid construction). Protein expression was induced with 1 mmol/l IPTG shaking at 37 °C overnight. Then, cells were harvested and lysed on ice by sonication. The cell lysis was centrifuged at 12,000 ×*g* for 5 min at 4 °C. The supernatants and sediments were collected and detected for protein expression by SDS-PAGE. GST-GdDnaJ and His-RdRp fusion proteins were purified using Ni-agarose resin (ComWin Biotech) and GST-agarose resin (ComWin Biotech), respectively, according to the manufacturer’s instructions. The GST pull-down assay was performed as previously described [22].

### Co-immunoprecipitation

HEK-293 T cells were transfected with pcDNA3.1-Myc-His-RdRp and pcDNA3.1-HA-GdDnaJ constructs (see above, plasmid construction) using Lipofectamine 2000 (Invitrogen). Cells transfected with pcDNA3.1-Myc-His-RdRp + pcDNA3.1-HA empty vector and pcDNA3.1-Myc-His empty vector + pcDNA3.1-HA-GdDnaJ were used as controls. After transfection for 40 h, the cells were lysed in RIPA lysis buffer (Biosharp) with PMSF (Sigma-Aldrich). Then, the cell lysates were clarified by centrifugation at 12,000 ×*g* for 15 min at 4 °C. The supernatant was incubated overnight with anti-His antibody (mouse, TransGen Biotech) at 4 °C. Subsequently, the mixtures were incubated with protein A/G-Sepharose beads (Sangon Biotech) for an additional 4 h at 4 °C. The immune precipitates were collected by centrifugation at 500 ×*g* for 2 min and were washed with lysis buffer three times. The interaction between RdRp and GdDnaJ was detected by SDS-PAGE and western blot analysis with an anti-HA antibody (mouse, TransGen Biotech). The reciprocal immunoprecipitation was performed in the same way using the anti-HA antibody for precipitation, followed by detection of the interaction via the anti-His antibody.

### Bimolecular fluorescence complementation (BiFC) assay

pbFos-RdRp and pbJun-GdDnaJ plasmids (see above, plasmid construction) were mixed at a ratio of 1:1 and co-transfected into HEK-293 T cells using Lipofectamine 2000 transfection reagents (Thermo Fisher Scientific). Cells co-transfected with pbJun-KN151 and pbFos-LC151 plasmids were used as positive control, and those co-transfected with pHA-KN151 and pMyc-LC151 plasmids were used as negative control. Cells were then incubated at 37 °C (with 5% CO_2_) for 24 h and immobilized by paraformaldehyde for 10 min. DAPI was used to stain the nucleus. The fluorescence was detected by using the 543 nm laser line of a laser scanning confocal microscope (FluoView FV1000, Olympus).

### Antibodies

To generate polyclonal antibodies against RdRp and GdDnaJ, pET-32a-RdRp and pGEX-4 T-1-GdDnaJ constructs (see above, plasmid construction) were expressed in Transetta (DE3) cells and purified as described above (see above, GST pull-down). Then, 6-month-old rabbits and 6-week-old Balb/c mice were immunized with 100 μg His-RdRp protein and 500–1000 μg GST-GdDnaJ protein three times, respectively. After the final injection, whole blood was collected and the serum was separated. The anti-*G. duodenalis* tubulin (Gdtubulin) polyclonal antibody was previously prepared in our laboratory.

### Duolink proximal ligation assay (Duolink PLA)

The Duolink PLA assays were performed using the Duolink In Situ Red Starter Kit Mouse/Rabbit (Sigma-Aldrich) as previously described [23]. Anti-RdRp (rabbit) and anti-GdDnaJ (mouse) antibodies were used for this assay. The fluorescence was detected by confocal microscopy (FluoView FV1000, Olympus).

### Functional study of GdDnaJ

*Giardia* trophozoites were collected by centrifugation. After counting, the trophozoites were inserted into fresh *Giardia* medium at 1 × 10^6^ trophozoites/tube. After incubation at 37 °C for 6 h, GdDnaJ inhibitor KNK437 (Selleck) was added to the medium at concentrations of 25 μM, 50 μM, 75 μM, 100 μM, 125 μM and 150 μM, with three replicates of each concentration. PBS and DMSO were set as controls. Incubation was continued at 37 °C for 18 h, and trophozoites were collected. After counting, lysis buffer was used to lyse the same number (5 × 10^6^ trophozoites) of *Giardia* trophozoites collected at different inhibitor concentrations. SDS-PAGE samples were prepared after ice bath and ultrasonic fragmentation. The appropriate concentration of the KNK437 inhibitor to inhibit GdDnaJ expression was verified by western blot.

### Quantitative real-time PCR

To assess the influence of KNK437 on GLV replication, we chose a KNK437 concentration that cannot affect the *Giardia* vitality to treat the same number of *Giardia* trophozoites (5 × 10^6^ trophozoites). Total RNA of *Giardia* trophozoites was isolated using Trizol (Invitrogen) and reverse-transcribed into cDNA, which was used as the template for qPCR. The data of GLV capsid protein mRNA were normalized to *G. duodenalis* actin (Gdactin). The expression level of GLV was determined by quantitative real-time PCR analysis using a SYBR Green PCR kit (Roche). Gene expression levels were calculated by the comparative CT method. The primers used for qPCR are listed in Additional file 1: Table S2.

### Statistical analysis

Statistical significance was determined at the *P* < 0.05 (*), *P* < 0.01 (**), *P* < 0.001 (***) and *P* < 0.0001 (****) levels by Student’s *t-test* using GraphPad Prism 6 software.

## Results

### Identification of GdDnaJ as a novel binding protein of RdRp

To identify proteins that can interact with GLV RdRp, we screened the *Giardia* cDNA library with amino acids 452–690 of RdRp as the bait using Y2H screen. Approximately 3.7 × 10^6^ clones were screened and 125 positive candidate clones identified (our unpublished data). BLAST (Basic Local Alignment Search Tool) analysis revealed that these candidate clones encode 37 unique proteins. The subsequent return verification analysis identified that only three proteins were potential interacting partners of RdRp (our unpublished data). Intriguingly, one of them was *Giardia* chaperone protein GdDnaJ. The interaction between RdRp and GdDnaJ was preliminarily verified by alpha-galactosidase assay.

### In vitro and ex vivo analysis of the interaction between GdDnaJ and RdRp

**Fig. 1:**
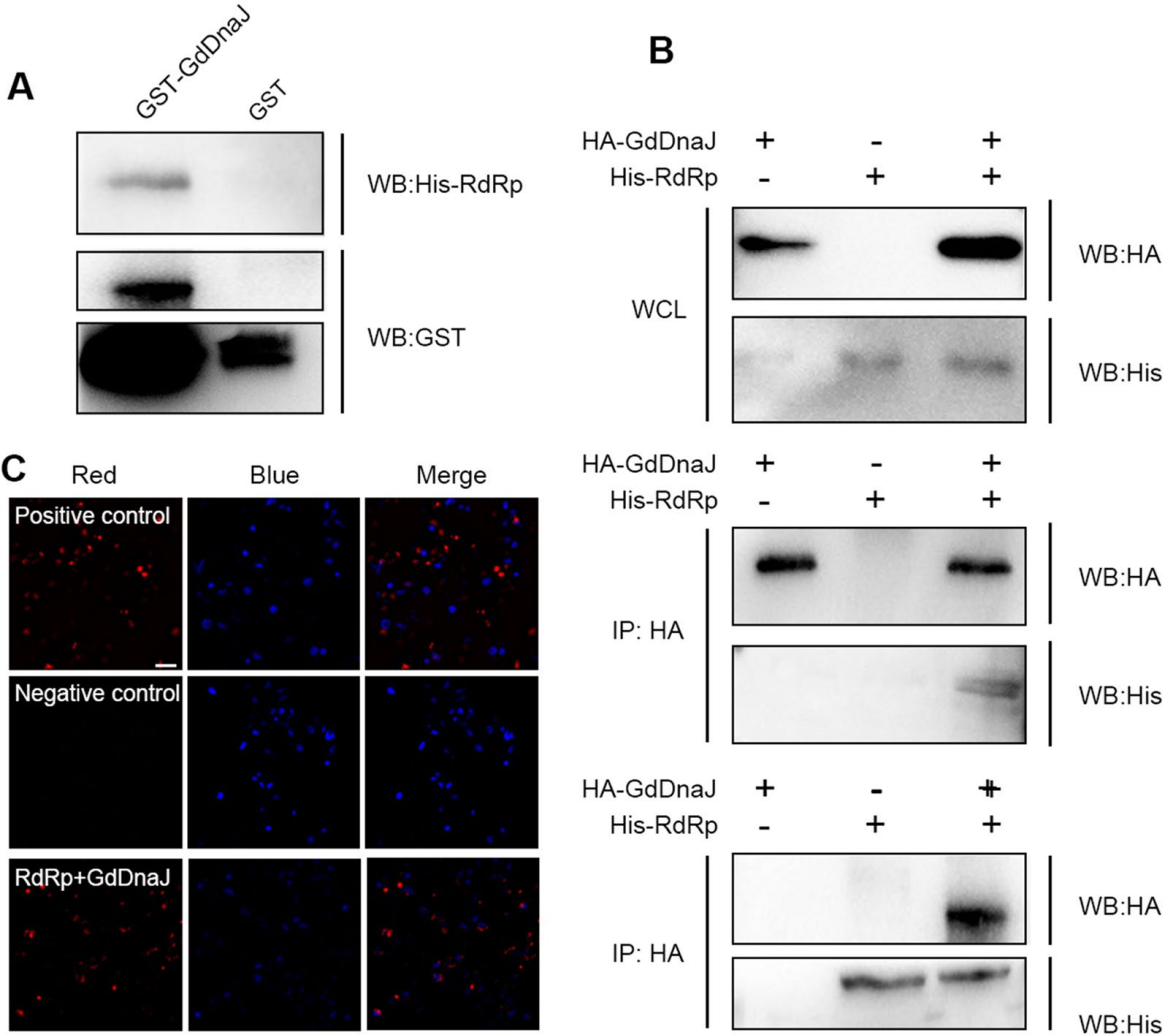
In vitro and ex vivo analysis of the interaction between RdRp and GdDnaJ. **A** GST pull-down assay showed a direct interaction between RdRp and GdDnaJ in vitro. **B** Co-immunoprecipitation analysis showed reciprocal binding of endogenous RdRp and GdDnaJ in HEK293T cell lysates. **C** BiFC assay confirms the direct interaction between RdRp and GdDnaJ in HEK293T cells. Cells co-transfected with pbJun-KN151 and pbFos-LC151 and pHA-KN151 and pMyc-LC151 were used as positive and negative control, respectively. The nuclei stained by DAPI are shown in blue. Scale bar: 50 μm

To verify the interaction between GdDnaJ and RdRp, GST pull-down assay was performed. The results showed that GST-GdDnaJ, but not GST alone, was able to pull down His-tagged RdRp, indicating a direct physical interaction between GdDnaJ and RdRp in vitro (Fig. 1A). In addition, we performed a co-immunoprecipitation experiment in HEK-293 T cells to see if GdDnaJ can interact with RdRp. The HA antibody was able to pull down His-RdRp only when both His-RdRp and HA-GdDnaJ were expressed in the HEK-293 cells, supporting the interaction of these two proteins ex vivo (Fig. 1B). To further analyze the association between RdRp and GdDnaJ, we employed BiFC assay in HEK293T cells. As expected, the cells containing both pbFos-RdRp and pbJun-GdDnaJ, but not those containing the empty vectors pHA-KN151 and pMyc-LC151, generated a fluorescent signal in the cytoplasm of HEK293 cells, consistent with a specific association between GdDnaJ and RdRp (Fig. 1C). Taken together, these results support a direct physical interaction between GdDnaJ and RdRp both in vitro and in live cells ex vivo.

### Colocalization and in vivo interaction of GdDnaJ and RdRp in *Giardia trophozoites*

**Fig. 2:**
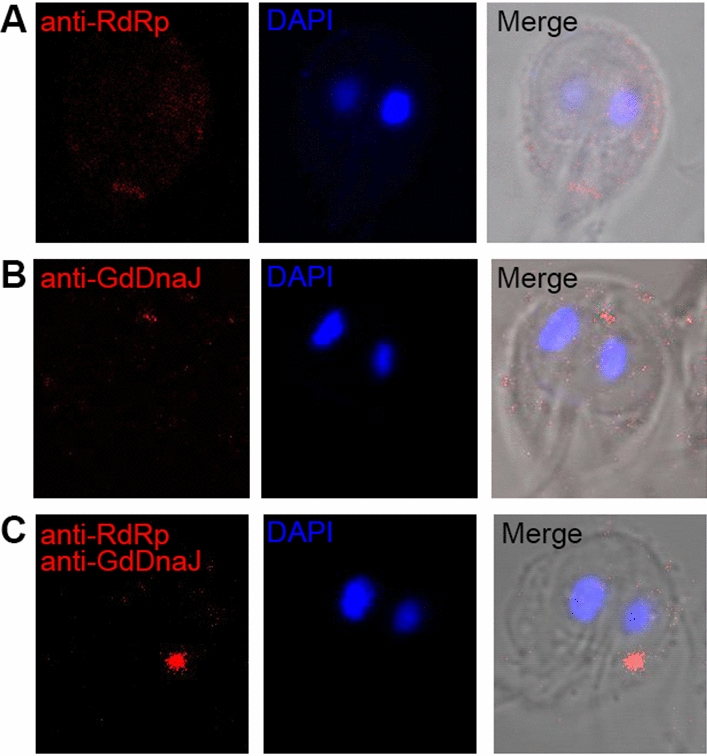
Colocalization and in vivo interaction of GdDnaJ and RdRp in *Giardia* trophozoites. Duolink PLA assay and immunofluorescence detect the colocalization and interaction between RdRp and GdDnaJ. **A** Localization of RdRp in *Giardia* trophozoites (in red). **B** Localization of GdDnaJ in *Giardia* trophozoites (in red). **C** Red fluorescence shows colocalization between GdDnaJ and RdRp in *Giardia* trophozoites. The nuclei of *Giardia* stained with DAPI are shown in blue. Anti-RdRp (rabbit) and anti-GdDnaJ (mouse) antibodies were used

To further validate the interaction between GdDnaJ and RdRp in vivo, Duolink PLA was used to examine the colocalization and association of GdDnaJ and RdRp in *Giardia* trophozoites. Red fluorescence was found in the cytoplasm of *Giardia*, indicating that GdDnaJ can interact with RdRp in vivo in *Giardia* trophozoites (Fig. 2).

### GdDnaJ affected the proliferation of *Giardia* and replication of GLV

**Fig.3:**
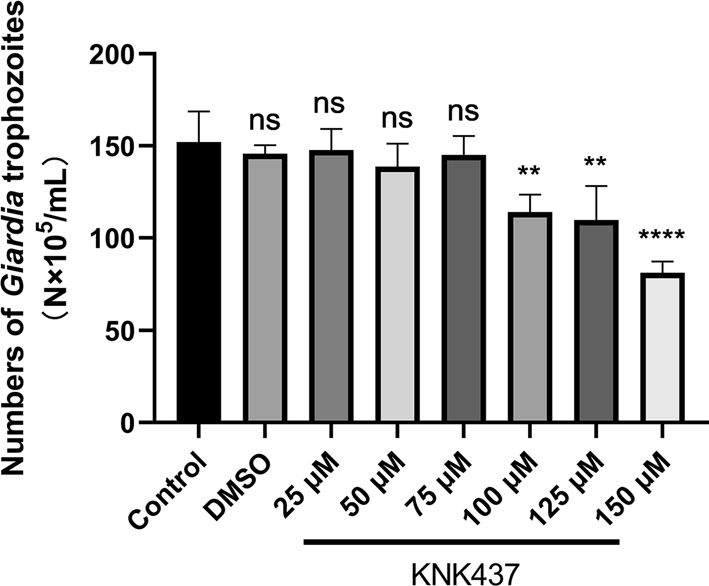
Effects of KNK437 on *Giardia* proliferation. *Giardia* trophozoite counting showed that KNK437 affected *Giardia* proliferation in a dose-dependent manner (25 μM, 50 μM, 75 μM, 100 μM, 125 μM and 150 μM) after incubating at 37 °C for 18 h, with three replicates of each concentration. PBS and DMSO (KNK437 solvent) were set as controls. Results were expressed as mean ± SD from three separate experiments. *ns* no significant difference, *P < 0.05, **P < 0.01, ***P < 0.001, ****P < 0.0001

**Fig.4:**
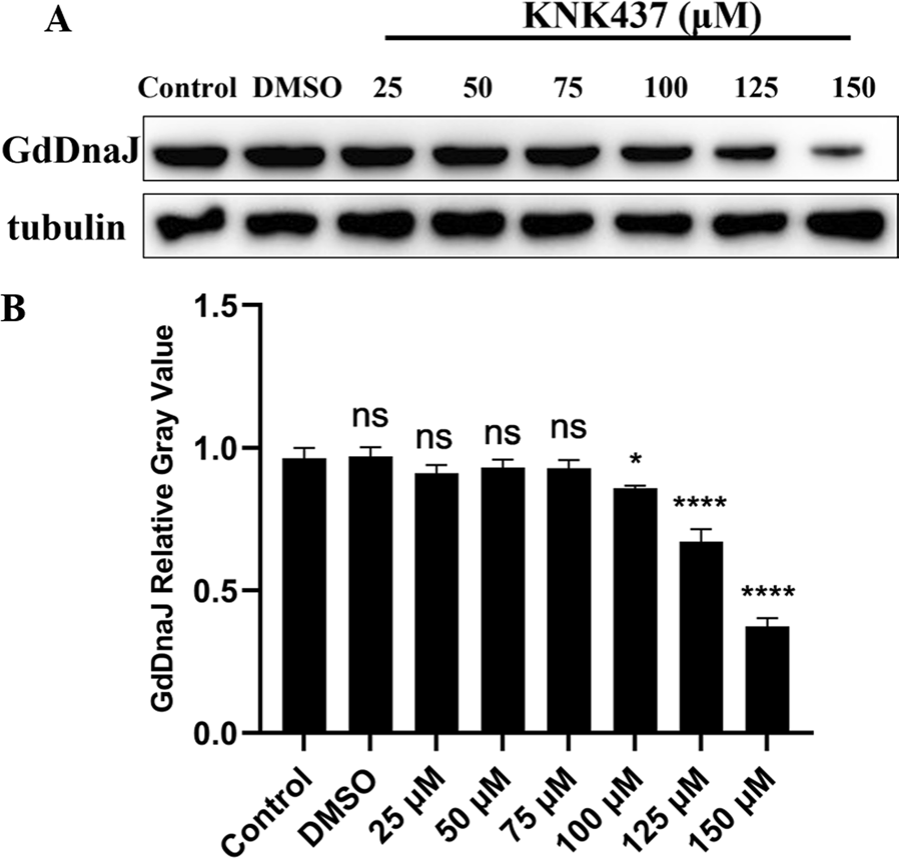
Effects of KNK437 on the expression of GdDnaJ. **A** Protein expressions of GdDnaJ by treatment with KNK437 at different concentrations (25 μM, 50 μM, 75 μM, 100 μM, 125 μM and 150 μM) were measured by western blot. Gd tubulin was the control. **B** Relative gray values of GdDnaJ are shown. Results are expressed as mean ± SD from three separate experiments. *ns* no significant difference, *P < 0.05, **P < 0.01, ***P < 0.001, ****P < 0.0001

**Fig.5:**
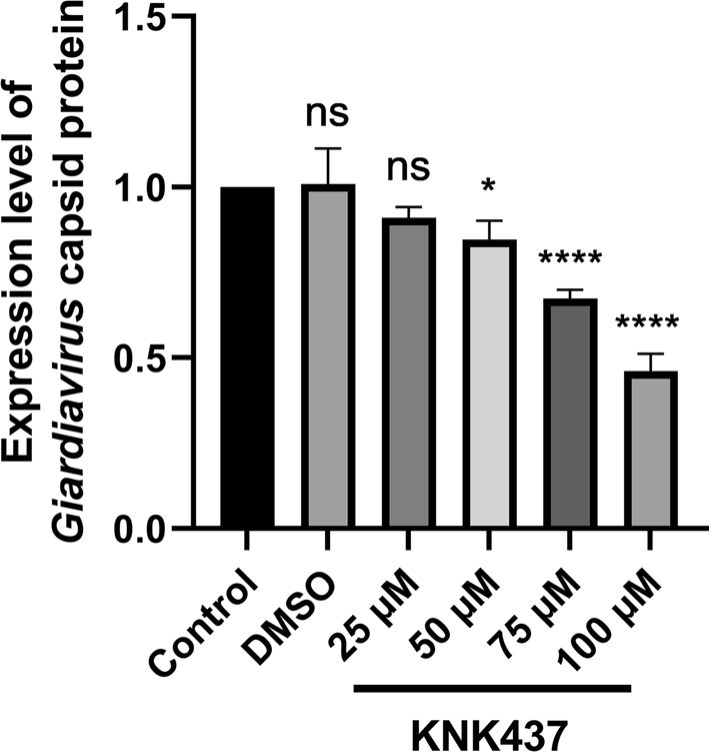
Effects of KNK437 on the GLV expression level. The expression level of GLV capsid protein treated with KNK437 at different concentrations (25 μM, 50 μM, 75 μM and 100 μM) was measured by qPCR, with three replicates of each concentration. The data of GLV capsid protein mRNA were normalized to Gd actin. The cDNA concentration of *Giardia* trophozoites was the same in each group. Primers of qPCR are listed in Additional file 1: Table S2. Results are expressed as mean ± SD from three separate experiments. ns, no significant difference, *P < 0.05, **P < 0.01, ***P < 0.001, ****p < 0.0001

To explore the function of chaperone protein GdDnaJ, we used KNK437, a pan-HSP (heat shock proteins) inhibitor, to detect the effects of GdDnaJ on *Giardia* proliferation and GLV replication. By counting the number of *Giardia* trophozoites after treatment with different KNK437 concentrations, we found that KNK437 concentrations < 75 μM had no significant effect on the proliferation of trophozoites (Fig. 3). Similarly, we found there was no significant difference in GdDnaJ expression by western blot, especially if the KNK437 concentration was < 75 μM (*P < 0.05) (Fig. 4A, B). Furthermore, the trophozoite numbers decreased with increasing KNK437 concentrations (> 75 μM), which indicated that the *Giardia* proliferation could be significantly affected by inhibiting GdDnaJ expression. Interestingly, by detecting the expression levels of GLV capsid protein through qPCR (Fig. 5), we found that they were significantly inhibited at concentrations of 50 μM and 75 μM, and the expression decreased significantly with increasing KNK437 concentration, suggesting that the inhibition of GLV replication by KNK437 might not be influenced by the reduction in *Giardia* proliferation (Fig. 5). Taken together, these results implied that GdDnaJ could influence *Giardia* proliferation and affect GLV replication at appropriate KNK437 concentrations (75–100 μM).

## Discussion

So far, a few regulatory proteins of RdRp in dsRNA virus have been reported. It has been shown that the activity of RdRp in RNA virus needs the participation of host factors [24]. In the replicase complex of tomato cluster dwarf virus, the activity of RdRp replicase needs the activation of host factor heat shock protein 70, which indicates that the function of RdRp needs the regulation of host regulatory protein [25]. The interaction between RdRp and OTU-like cysteine enzyme in *Eimeria tenella* virus can enhance the activity of cysteine deubiquitinating enzyme [26]. In addition, the activity of RdRp in *Leishmania* virus was decreased when the density of *Leishmania* was increased, which indicates that there is a correlation between the proliferation of *Leishmania* and *Leishmania* virus replication [27]. However, little is known about the regulator protein of RdRp in GLV. As *Giardia* is a good model organism for studying the relationship between host and virus, revealing the regulatory mechanism of GLV is of great significance for elucidating the regulatory mechanism of dsRNA virus in other higher animals. In this study, we performed a Y2H screen and identified the chaperone protein, GdDnaJ, as a new interacting partner of GLV RdRp. This direct interaction was confirmed both in vitro and ex vivo in human cell lines through GST pull-down, co-immunoprecipitation and BiFC assays. In addition, Duolink PLA verified the colocalization and interaction between GdDnaJ and RdRp in *Giardia* trophozoites, suggesting a correlation of these two proteins in vivo.

DnaJ, also known as Hsp40 (heat shock protein 40), belongs to the J-domain protein family members [28]. It has been shown that DnaJ can activate ATPase mainly by stimulating hsp70s. DnaJ plays an important role in protein translation, folding and degradation. However, the function of GdDnaJ in *Giardia* has not been explicitly explored. KNK437 is a widely used HSP inhibitor, which can inhibit the activity of Hsp40, Hsp70 and Hsp105. It affects the interaction between heat shock factor HSF and HSE and inhibits the transcription of heat shock protein mRNA, thus down regulating the expression of HSP proteins [29]. In addition, KNK437 can inhibit the production of virus particles during the baculovirus infection cycle [30]. To explore the potential role of DnaJ in regulating the activity of GLV RdRp protein, a virus-mediated hammerhead ribozyme and protein inhibitors can be used [31]. We here treated *Giardia* trophozoites with KNK437 and observed the effect of DnaJ on the proliferation of *Giardia* and the replication of GLV. Our results showed that different concentrations of KNK437 could obviously inhibit the proliferation of *Giardia* and reduce the replication of GLV, which indicated that the activity of RdRp enzyme was decreased after KNK437 treatment, suggesting that there might be positive regulation between GdDnaJ and GLV RdRp protein.

In conclusion, our results identified GdDnaJ as a new binding partner of GLV RdRp. In addition, GdDnaJ could potentially regulate the proliferation of *Giardia* and the replication of GLV via binding with RdRp.

## Supplementary Information

**Additional file 1: Table S1.** Primers used for plasmid construction. **Table S2.** Primers used for quantitative real-time PCR.

## Data Availability

The data generated and analyzed during this study are included in the article.

## Abbreviations

BiFC: Bimolecular fluorescence complementation
GdDnaJ: *Giardia* DnaJ protein
Duolink PLA: Duolink proximal ligation assay
RdRp: RNA-dependent RNA polymerase
Y2H: Yeast two-hybrid

## References

[1] Dixon BR (2021). *Giardia duodenalis* in humans and animals—Transmission and disease. Res Vet Sci.

[2] Heyworth MF (2016). *Giardia duodenalis* genetic assemblages and hosts. Parasite.

[3] Einarsson E, Ma'ayeh S, Svärd SG (2016). An up-date on *Giardia* and giardiasis. Curr Opin Microbiol.

[4] Torgerson PR, Devleesschauwer B, Praet N, Speybroeck N, Willingham AL, Kasuga F, Rokni MB, Zhou XN, Fèvre EM, Sripa B (2015). World Health Organization estimates of the global and regional disease burden of 11 foodborne parasitic diseases, 2010: a data synthesis. PLoS Med.

[5] Kirk MD, Pires SM, Black RE, Caipo M, Crump JA, Devleesschauwer B, Döpfer D, Fazil A, Fischer-Walker CL, Hald T (2015). World Health Organization estimates of the global and regional disease burden of 22 foodborne bacterial, protozoal, and viral diseases, 2010: a data synthesis. PLoS Med.

[6] Ankarklev J, Jerlström-Hultqvist J, Ringqvist E, Troell K, Svärd SG (2010). Behind the smile: cell biology and disease mechanisms of *Giardia* species. Nat Rev Microbiol.

[7] Savioli L, Smith H, Thompson A (2006). Giardia and Cryptosporidium join the 'neglected diseases initiative'. Trends Parasitol.

[8] Halliez MC, Buret AG (2013). Extra-intestinal and long term consequences of *Giardia duodenalis* infections. World J Gastroenterol.

[9] Luján H, Svrd S (2011). *Giardia*: a model organism.

[10] Adam RD (2001). Biology of *Giardia lamblia*. Clin Microbiol Rev.

[11] Carranza PG, Lujan HD (2010). New insights regarding the biology of *Giardia lamblia*. Microbes Infect.

[12] Cai W, Ryan U, Xiao L, Feng Y (2021). Zoonotic giardiasis: an update. Parasitol Res.

[13] Gómez-Arreaza A, Haenni AL, Dunia I, Avilán L (2017). Viruses of parasites as actors in the parasite-host relationship: a "ménage à trois". Acta Trop.

[14] Barrow P, Dujardin JC, Fasel N, Greenwood AD, Osterrieder K, Lomonossoff G, Fiori PL, Atterbury R, Rossi M, Lalle M (2020). Viruses of protozoan parasites and viral therapy: is the time now right?. Virol J.

[15] Ives A, Ronet C, Prevel F, Ruzzante G, Fuertes-Marraco S, Schutz F, Zangger H, Revaz-Breton M, Lye LF, Hickerson SM (2011). Leishmania RNA virus controls the severity of mucocutaneous leishmaniasis. Science.

[16] Fichorova RN, Lee Y, Yamamoto HS, Takagi Y, Hayes GR, Goodman RP, Chepa-Lotrea X, Buck OR, Murray R, Kula T (2012). Endobiont viruses sensed by the human host—beyond conventional antiparasitic therapy. PLoS ONE.

[17] Wang AL, Wang CC (1986). Discovery of a specific double-stranded RNA virus in *Giardia*
*lamblia*. Mol Biochem Parasitol.

[18] Wang AL, Yang HM, Shen KA, Wang CC (1993). Giardiavirus double-stranded RNA genome encodes a capsid polypeptide and a gag-pol-like fusion protein by a translation frameshift. Proc Natl Acad Sci USA.

[19] Lagunas-Rangel FA, Kameyama-Kawabe LY, Bermúdez-Cruz RM (2021). Giardiavirus: an update. Parasitol Res.

[20] Tai JH, Ong SJ, Chang SC, Su HM (1993). Giardiavirus enters *Giardia*
*lamblia* WB trophozoite via endocytosis. Exp Parasitol.

[21] Gong P, Li X, Wu W, Cao L, Zhao P, Li X, Ren B, Li J, Zhang X (2020). A novel MicroRNA from the translated region of the *Giardiavirus* rdrp gene governs virus copy number in *Giardia*
*duodenalis*. Front Microbiol.

[22] Zhang N, Wang X, Gobel V, Zhang X (2018). The galectin LEC-5 is a novel binding partner for RAB-11. Biochem Biophys Res Commun.

[23] Li X, Zhang N, Wu N, Li J, Yang J, Yu Y, Zheng J, Li X, Wang X, Gong P (2020). Identification of GdRFC1 as a novel regulator of telomerase in *Giardia*
*duodenalis*. Parasitol Res.

[24] Ahlquist P (2002). RNA-dependent RNA polymerases, viruses, and RNA silencing. Science.

[25] Pogany J, Nagy PD (2015). Activation of tomato bushy stunt virus RNA-dependent RNA polymerase by cellular heat shock protein 70 Is enhanced by phospholipids in vitro. J Virol.

[26] Wang P, Li J, Gong P, Wang W, Ai Y, Zhang X (2018). An OTU deubiquitinating enzyme in Eimeria tenella interacts with *Eimeria*
*tenella* virus RDRP. Parasit Vectors.

[27] Weeks RS, Patterson JL, Stuart K, Widmer G (1992). Transcribing and replicating particles in a double-stranded RNA virus from Leishmania. Mol Biochem Parasitol.

[28] Walsh P, Bursać D, Law YC, Cyr D, Lithgow T (2004). The J-protein family: modulating protein assembly, disassembly and translocation. EMBO Rep.

[29] Ohnishi K, Takahashi A, Yokota S, Ohnishi T (2004). Effects of a heat shock protein inhibitor KNK437 on heat sensitivity and heat tolerance in human squamous cell carcinoma cell lines differing in p53 status. Int J Radiat Biol.

[30] Lyupina YV, Dmitrieva SB, Timokhova AV, Beljelarskaya SN, Zatsepina OG, Evgen'ev MB, Mikhailov VS (2010). An important role of the heat shock response in infected cells for replication of baculoviruses. Virology.

[31] Chen L, Li J, Zhang X, Liu Q, Yin J, Yao L, Zhao Y, Cao L (2007). Inhibition of krr1 gene expression in *Giardia canis* by a virus-mediated hammerhead ribozyme. Vet Parasitol.

