# Supplementary material for: A potential role for Giardia chaperone protein GdDnaJ in regulating Giardia proliferation and Giardiavirus replication
Source: Parasit Vectors. 2023 May 25;16:168. doi: 10.1186/s13071-023-05787-0 (PMC10210397; doi:10.1186/s13071-023-05787-0)
Supplement: Supplementary file 1 — Additional file 1: Table S1. Primers used for plasmid construction. Table S2. Primers used for quantitative real-time PCR. [file 13071_2023_5787_MOESM1_ESM.docx]

**Table S1. Primers used for plasmid construction**

| **Primer name** | **Primer sequence (5’-3’)** |
| --- | --- |
| pGBKT7-RdRp-F | *CATATG*GATAATCCACCCCTCAAGC |
| pGBKT7-RdRp-R | *GTCGAC*AATCATCATTCTAGCTGGGTAC |
| pET-32a-RdRp-F | *GGATCC*GATAATCCACCCCTCAAGC |
| pET-32a-RdRp-R | *AAGCTT*AATCATCATTCTAGCTGGGTA |
| pGEX-4T-1-GdDnaJ-F | CCG*GAATTC*ATGGGTAGGAGTTTCTATGAGG |
| pGEX-4T-1-GdDnaJ-R | CCG*CTCGAG*ATTTGGCGGCAGCAGCTCCA |
| pcDNA-His-RdRp-F | CGC*GGATCC*GCCACCATGGATAATCCACCCCTCAAGC |
| pcDNA-His-RdRp-R | ATAAGAAT*GCGGCCGC*AATCATCATTCTAGCTGGGTA |
| pcDNA-HA-GdDnaJ-F | CTA*GCTAGC*CACCATGGGTAGGAGTTTCTATGAGG |
| pcDNA-HA-GdDnaJ-R | CCG*CTCGAG*ATTTGGCGGCAGCAGCTCCA |
| pbFos-RdRp-F | *GCTAGC*CACCATGGATAATCCACCCCTCAAGC |
| pbFos-RdRp-R | *CGATCG*AAATCATCATTCTAGCTGGGTAC |
| pbJun-GdDnaJ-F | *GCTAGC*CACCATGGGTAGGAGTTTCTATGAGG |
| pbJun-GdDnaJ-R | *CTCGAG*CTATTTGGCGGCAGCAGCTCCA |

The restriction enzyme sequences are underlined.

**Table S2. Primers used for Quantitative real-time PCR**

| **Primer name** | **Primer sequence (5’-3’)** |
| --- | --- |
| Capsid-F | GCTTTTGCCCTCGTCTACC |
| Capsid-R | ATCCCACACGCTCTTGACTT |
| *Giardia*actin-F | CAGAACTGGCGTCAAACGTG |
| *Giardia*actin-R | TTTCCTCCATACCACACGGC |
